# Sex Differences in Effects of Mood, Eating-Related Behaviors, and BMI on Food Appeal and Desire to Eat: A Cross-Sectional Survey Study

**DOI:** 10.3390/nu15030762

**Published:** 2023-02-02

**Authors:** Kristina T. Legget, Marc-Andre Cornier, Lauren Sarabia, Eve M. Delao, Susan K. Mikulich-Gilbertson, Crystal Natvig, Christina Erpelding, Tessa Mitchell, Allison Hild, Eugene Kronberg, Jason R. Tregellas

**Affiliations:** 1 Department of Psychiatry, School of Medicine, University of Colorado Anschutz Medical Campus, Aurora, CO 80045, USA; 2 Research Service, Rocky Mountain Regional VA Medical Center, Aurora, CO 80045, USA; 3 Division of Endocrinology, Diabetes and Metabolic Diseases, Department of Medicine, Medical University of South Carolina, Charleston, SC 29425, USA

**Keywords:** eating behaviors, sex differences, visual food cues, appetite, mood

## Abstract

Although sex differences in food intake have been observed consistently, contributing factors are not well understood. Using a cross-sectional online survey (*n* = 306; 151 men, 155 women), this study aimed to assess how sex impacts relationships between food ratings (appeal/desire to eat for high-calorie (HC) and low-calorie (LC) food images) and eating-related attitudes/behaviors, body mass index (BMI), and mood. Across participants, increased state- and trait-based hunger, disinhibition, and cravings were associated with both increased HC appeal and desire (*p* < 0.001). Increased state-based hunger and cravings were associated with greater LC desire (*p* < 0.001). Greater satiety was associated with decreased desire for both HC and LC (*p* < 0.001), while greater anxiety was associated with increased desire for both HC and LC (*p* < 0.001). Significant associations between BMI and food ratings were not observed. Women reported greater dietary restraint, trait-based hunger, disinhibition, eating disorder-related behaviors, depression, and stress compared to men, in addition to greater appeal and familiarity with LC foods (all *p* < 0.05). Significant effects of sex on the associations between food ratings and eating-related attitudes/behaviors, BMI, and mood were not observed, however. Findings support the importance of considering mood and eating-related attitudes/behaviors in investigations of food cue responsivity.

## 1. Introduction

Obesity rates are rapidly rising [1,2,3], contributing to substantial impacts on health and quality of life [1,4,5]. As such, gaining a better understanding of the mechanisms involved in the development and maintenance of obesity is critical. Because eating behaviors are core contributors to weight gain [6,7], an improved understanding of these processes is required. A key variable that is currently poorly understood is the effect of sex. Sex-based differences in eating behaviors have been consistently observed, with women consuming more fruits and vegetables, reporting a greater emphasis on healthy eating, being more likely to engage in dieting, reporting greater body dissatisfaction, and engaging in more eating disorder-related behaviors [8,9,10,11,12]. Multiple potential factors underly these differences, including expression of gonadal and appetite-related hormones, hypothalamic-pituitary-adrenal (HPA) axis signaling, reward processing, and physical activity engagement [8,13,14,15]. Another factor is the impact of societal norms and pressures regarding food and weight, with pressures for men to pursue a muscular physique and women to pursue a thin physique [16], along with expectations that women should eat a healthier diet and less food compared to men [17]. These pressures can contribute to guilt, stress, and anxiety regarding food choices and consumption. Despite reported sex differences in eating behaviors, however, the majority of studies investigating the roles of behavior and food preferences in weight-gain and obesity fail to appropriately consider the impact of sex. Examining which factors may differentially impact food intake behaviors in men and women will be key in understanding mechanisms underlying weight gain and obesity, for improving reproducibility of pre-clinical and clinical studies, and for the successful development of individualized weight management approaches [18].

A common approach to studying mechanisms underlying food intake behaviors is to measure responsivity to visual food cues (e.g., brain response [19,20,21,22]; attentional bias [23,24]). How sex impacts responsivity to visual food cues, however, is unclear. Neuroimaging studies have observed sex-based differences in brain responsivity to food cues, but results have been mixed [14,25,26,27,28,29]. In addition to the limited sample sizes in these studies, differences in variables such as BMI or satiety level across study cohorts also likely contribute to variability in findings [14,25]. A first step in better understanding this is to determine how sex differences affect attitudes towards visual food cues (e.g., appeal, desire to eat) and how effects of sex influence other contributing factors towards these attitudes. As such, the goal of the current study was to investigate how sex impacts (a) ratings of food appeal and desire to eat, for both high- and low-calorie visual food cues, and (b) key contributing factors to those ratings. Specifically, we assessed effects of sex on relationships between ratings of food appeal/desire to eat and eating behaviors/attitudes (state-based hunger and satiety; trait-based hunger, dietary restraint, and disinhibition; food cravings; eating disorder-related behaviors), along with mood (depression, anxiety, and stress) and BMI. Ratings of appeal targeted stable attitudes towards foods (i.e., “How appealing is this food, in general?”), while ratings of desire to eat targeted state-based attitudes (i.e., “How much do you desire to eat this food, right now?”) [30].

Given increased rates of eating disorder-related behaviors in women [10,31], we hypothesized that these behaviors would be more prevalent in women compared to men and, as such, would have a greater impact on ratings of both appeal and desire in women, particularly for high-calorie (HC) foods. Based on previous studies, we also anticipated higher rates of dietary restraint in women [26,30] and that higher levels of restraint would be associated with reduced desire to eat HC foods. As previous studies have found emotional eating (i.e., eating in response to negative affective states) to be associated with increased HC food craving and consumption and that women report higher rates of emotional eating compared to men [32,33], we hypothesized that mood measures (depression, anxiety, stress) would have a greater impact on food ratings in women compared to men. We also hypothesized that BMI would be associated with higher ratings of high-calorie appeal and desire, in concordance with findings from a previous study by our group that assessed effects of portion size on food appeal and desire to eat, using a similar set of food images [30]. Neuroimaging studies have also identified associations between brain response to food cues and both BMI [34,35,36,37] and subsequent weight gain [38,39]. How sex impacts the effects of BMI on visual food cue responsivity is not well understood [14,40]. However, as a previous study assessing ratings of “liking” foods presented as written words (e.g., “burger”) found a greater difference between BMI categories (normal-weight, overweight, or obese) in liking ratings of high-fat foods in women than men [41], we hypothesized that the relationship between BMI and high-calorie appeal and desire ratings for visual food cues would be more prominent in women than men. We were also interested in examining how discrepancies between HC and LC ratings may be impacted by these factors. An overall preference for HC compared to LC foods may serve as a useful indicator of food attitudes; i.e., if both HC and LC foods are rated highly, it is likely easier to select LC foods than if HC foods are consistently perceived as more appealing and desirable than LC foods. As such, we hypothesized that greater HC–LC discrepancies would be associated with increased BMI, as well as increased hunger, disinhibition, and cravings, and that these associations would be more prominent for women than men.

## 2. Materials and Methods

### 2.1. Participants and Recruitment

Participants were recruited by referral and targeted Facebook and Twitter advertisements from March 2020 to December 2020. For Facebook advertisements, a multistage sampling strategy was used to solicit responses from areas not typically included in academic research studies (i.e., outside university cities). We explicitly stratified the advertising by five geographic regions (northeast, southeast, midwest, northwest, and southwest). Implicit stratification was achieved through random selection of both a metropolitan and non-metropolitan area zip code within each region. Metropolitan zip codes included over 20,000 residents and either had a designation as part of a metropolitan statistical area or were at least the second most populated city in the state, with neighboring population centers. Non-metropolitan areas had fewer than 20,000 residents and were at least 25 miles from the nearest potential metropolitan area. For Twitter advertisements, the target group was initially “United States,” but an additional target group defined as “men” was added during the final stages of recruitment, to balance distribution by sex. Exclusion criteria included: (1) reporting a visual disability that would affect the ability to differentiate colors, (2) reporting a developmental impairment that could impact the ability to complete the study measures, and (3) reporting being pregnant. Participants provided written informed consent. All procedures were performed in accordance with and approved by the Colorado Multiple Institutional Review Board. A total of 333 adults (164 men, 169 women) completed the study, with 306 (151 men, 155 women) included in the analyses (see Section 2.5 for details on participants excluded from analyses).

### 2.2. Study Design

Participants completed the study online via REDCap (Research Electronic Data Capture [42,43], hosted at the University of Colorado Anschutz Medical Campus). Eligible participants were instructed to complete the survey in a neutral state of hunger. This was defined as having eaten something within the three hours before completing the assessments, but not within the two hours prior to participation (e.g., if completing the assessments at 2 pm, they should have eaten something between 11 am and noon, but not between noon and 2 pm). An infographic was used to help convey this instruction to participants (see Supplementary Figure S1). Although requested, this timing was not enforceable (mean reported time since eating = 3.0 ± 1.9 h; range 1.2–12.9 h). The study consisted of a battery of questionnaires and a food pictures task, both described below. Mean time to complete the food pictures task was 34.1 ± 10.8 min and completers were compensated with the choice of a US dollar (USD) 20 gift card to either Target or Amazon.

### 2.3. Questionnaires

Participants completed demographic questions including age, sex, race/ethnicity, and height/weight (used to calculate BMI). Participants then completed a series of eating- and mood-related questionnaires, in the following order: the Three Factor Eating Questionnaire (TFEQ), assessing trait-based eating behaviors relating to restraint, disinhibition, and hunger (score range for each subscale: restraint 0–21, disinhibition 0–16, hunger 0–14; higher scores indicate greater restraint, disinhibition, or hunger) [44]; the Eating Attitudes Test 26 (EAT-26), assessing eating disorder-related behaviors (score range: 0–78; higher scores indicate greater risk of disordered eating tendencies) [45]; the Center for Epidemiologic Studies Depression Scale-Revised (CESD-R), assessing symptoms associated with depression over the past week (score range: 0–60; higher scores indicate greater depressive symptoms) [46]; the Perceived Stress Scale (PSS), assessing feelings of stress over the past week (score range: 0–56; higher scores indicate greater perceived stress) [47]; the Food Cravings Questionnaire-State (FCQ-S), assessing state-based feelings relating to food cravings (score range: 15–75; higher scores indicate greater cravings) [48]; the State Trait Anxiety Inventory (STAI-state), assessing anxiety state (score range: 20–80; higher scores indicate greater anxiety) [49]; and visual analog scale (VAS) measures assessing current hunger (“how hungry are you?” from “not at all hungry” to “extremely hungry”) and satiety (“how full do you feel right now?” from “not at all full” to “extremely full”). VAS measures were scored on a 0–100 scale (with higher scores indicating greater hunger or satiety), but these numbers were not visible to participants. After these questionnaires were completed, participants began the food pictures task (description below).

### 2.4. Food Pictures Task

Participants were shown 96 different food images (48 high-calorie (HC) and 48 low-calorie (LC)) and asked to answer questions about each image, using REDCap. For this task, copyright-free images were selected from various websites, in addition to images from the International Affective Picture System (IAPS) database [50] and a pre-existing set of food images [30]. All images were sized to be 800 × 600 pixels. There were no differences in average image complexity (compression, spatial information, shade) or intensity between the HC and LC image sets (*p* > 0.05 for all). Number of calories (per 100 g) for each food image was estimated using the U.S. Department of Agriculture FoodData Central database (https://fdc.nal.usda.gov/ (accessed on 28 January 2021)). By design, average caloric content was significantly greater for the HC (mean ± standard deviation (SD): 323.94 ± 101.94 kcal) compared to LC image sets (mean ± SD: 59.19 ± 31.68 kcal), *t*(94) = 17.18, *p* < 0.001. Descriptions and caloric content for each of the food images can be found in Supplementary Table S1. Images were presented in the same randomized order for all participants. For each image, participants used a VAS to indicate their ratings of: (1) appeal (“How appealing is this food (in general)?” from “not appealing at all” to “extremely appealing”) and (2) desire to eat (“How much do you desire to eat this food (right now)?” from “no desire to eat this food” to “I have a strong desire to eat this food”). Responses were scored on a scale of 0–100 (with higher scores indicating greater appeal or desire to eat), but numerical values were not made visible to participants. Average ratings for appeal and desire to eat were calculated for both HC and LC foods for each participant. In addition to appeal and desire to eat, participants also rated each image for familiarity (“How familiar are you with this food?”), selecting one of four options for each image: (a) “I don’t recognize this food,” (b) “I recognize this food but have never tried it,” (c) “I recognize this food and have tried it before,” or (d) “I recognize this food and have had it often.” Familiarity ratings were scored from 1 (option a) to 4 (option d) and average familiarity scores were calculated for HC and LC foods for each participant.

### 2.5. Statistical Analyses

Distributions were assessed for outliers using the generalized Extreme Studentized Deviate test [51]. Two women were excluded from further analyses as outliers for BMI (BMI > 60 kg/m^2^) and 27 participants (12 men, 15 women) were excluded due to being outliers on time to complete the survey (>75 min). Demographic, questionnaire, and food task summary variables were compared between men and women using independent *t*-tests or chi-square tests, as appropriate (alpha of 0.05). Paired *t*-tests were used to assess across-group (across all participants) and within-group (separately for women and men) differences in food ratings (HC vs. LC, appeal vs. desire) and familiarity (HC vs. LC) (alpha of 0.05). The four variables generated from averaging desire and appeal ratings across high-calorie (HC) and low-calorie (HC) food image sets for each participant were separately modeled as outcome variables in multiple linear regressions to evaluate relationships with each a priori variable of interest (VAS measures of hunger and satiety; TFEQ restraint, hunger, and disinhibition; FCQ-S; EAT-26; CESD-R; STAI; PSS; BMI) and whether that relationship differed by sex. An example equation describing the model with interaction for the outcome of HC Appeal (Y_HC Appeal_) with VAS Hunger as the variable of interest is:

Y_HC Appeal_ = β_0_ + β_1_VAS Hunger + β_2_Sex + β_3_Sex ∗ VAS Hunger + β_4_Age + error (1)

Significance of β_3_ would indicate that the relationship between HC Appeal and VAS Hunger is different for men and women. An alpha of 0.05 was used to determine if this interaction term would remain in the model. If the *p*-value for the Sex*VAS Hunger interaction was larger than alpha = 0.05, it was removed and the model was re-fit as follows:

Y_HC Appeal_ = β_0_ + β_1_VAS Hunger + β_2_Sex + β_3_Age + error (2)

Significance of β_1_ in the reduced model would indicate that HC Appeal is related to VAS Hunger after adjusting for sex and age. Multiple linear regression results were corrected for multiple comparisons by increasing the statistical significance threshold to *p* = 0.002, reflecting a Bonferroni correction for an alpha of 0.05 (11 variables of interest and two types of food ratings (appeal, desire), for 22 comparisons). We similarly modeled relationships between the same variables of interest and the differences between HC and LC for both desire and appeal (variables created for HC–LC difference scores for both desire and appeal). Given group differences in age (see Table 1), all regressions were adjusted for age. All statistical analyses were performed using SAS 9.4 (SAS Institute, Cary, NC, USA) and R statistical software [52]. Unless otherwise indicated, data are expressed as means ± SD.

## 3. Results

### 3.1. Sex-Based Group Differences

As shown in Table 1, compared to men, women in the sample reported significantly greater dietary restraint, disinhibition, and trait-based hunger, as measured by the TFEQ. Significantly higher scores on the EAT-26 were also observed in women compared to men, as were significantly higher levels of perceived stress (from the PSS) and depression (from the CESD-R). Trends towards higher state-based anxiety (STAI) and higher levels of state-based hunger (VAS) were also observed in women compared to men, but did not reach statistical significance.

For food ratings, average familiarity scores (range of 1–4, with 4 indicating better familiarity than 1) suggested that participants were largely familiar with the presented foods (HC: 3.4 ± 0.3; LC: 3.5 ± 0.3). Sex differences in HC familiarity were not observed, but we did observe significantly greater LC food familiarity in women compared to men (see Table 1). Women also demonstrated a greater difference in HC vs. LC familiarity (with LC > HC) compared to men, *t*(304) = 5.80, *p* < 0.001. Across sex, ratings of food appeal were greater than ratings of desire to eat for both HC (*t*(305) = 18.91, *p* < 0.001) and LC (*t*(305) = 16.51, *p* < 0.001) foods, on average. Ratings of desire to eat HC or LC foods did not significantly differ by sex. Significant sex differences in HC food appeal were not observed, but men rated LC food appeal significantly lower than did women (Table 1). The difference between desire to eat HC vs. LC foods in women was significantly less than in men (Table 1), with no observed HC vs. LC desire difference in women (*t*(154) = −1.2, *p* = 0.238), but men rating HC foods as significantly more desirable than LC foods (*t*(150) = 2.55, *p* = 0.012). Similar group differences in LC vs. HC food *appeal* were observed (Table 1), with women rating HC and LC appeal similarly (*t*(154) = −0.94, *p* = 0.349), but men rating HC appeal as significantly higher than LC appeal (*t*(150) = 3.91, *p* < 0.001).

### 3.2. High-Calorie Food Ratings

**Figure 1:**
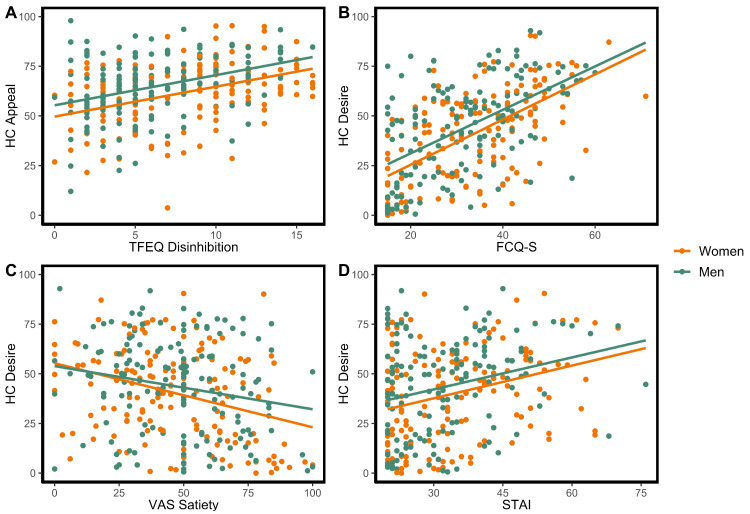
Representative selection of significant effects observed for associations with high-calorie (HC) food ratings (variables selected were ones in which significant effects were also observed for low-calorie (LC) food ratings, as shown in Figure 2). Significant associations were observed between: (**A**) HC appeal ratings (“Appeal”) and scores on the Three Factor Eating Questionnaire (TFEQ) disinhibition subscale (*F*(1,301) = 47.45, *p* < 0.001); (**B**) HC desire to eat ratings (“Desire”) and Food Cravings Questionnaire (FCQ-S) scores (*F*(1,301) = 137.24, *p* < 0.001); (**C**) HC desire to eat ratings and visual analog scale (VAS) ratings of satiety (*F*(1,301) = 20.32, *p* < 0.001); (**D**) HC desire to eat ratings and State-Trait Anxiety Inventory (STAI) scores (*F*(1,301) = 20.32, *p* < 0.001).

After adjusting for age and sex, higher ratings of both appeal and desire to eat HC food images were significantly associated with higher VAS hunger, greater disinhibition, greater trait-based hunger, and increased food cravings (Table 2; see Figure 1A,B). Higher ratings of desire to eat HC foods were associated with reduced VAS satiety (Figure 1C) and increased anxiety (Figure 1D), but this was not observed for ratings of appeal.

### 3.3. Low-Calorie Food Ratings

**Figure 2:**
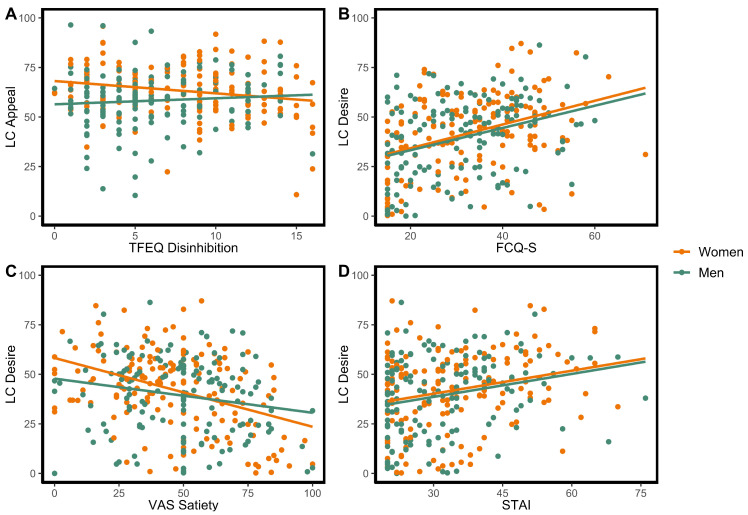
Representative selection of significant effects observed for associations with low-calorie (LC) food ratings (variables selected were ones in which significant effects were also observed for high-calorie (HC) food ratings, as shown in Figure 1). Significant associations were observed between: (**A**) LC appeal ratings (“Appeal”) and scores on the Three Factor Eating Questionnaire (TFEQ) disinhibition subscale (*F*(1,301) = 12.15, *p* < 0.001); (**B**) LC desire to eat ratings (“Desire”) and Food Cravings Questionnaire (FCQ-S) scores (*F*(1,301) = 40.66, *p* < 0.001); (**C**) LC desire to eat ratings and visual analog scale (VAS) ratings of satiety (*F*(1,301) = 29.25, *p* < 0.001); (**D**) LC desire to eat ratings and State-Trait Anxiety Inventory (STAI) scores (*F*(1,301) = 16.45, *p* < 0.001).

After adjusting for age and sex, a significant effect was observed for LC appeal ratings and disinhibition, potentially driven by increased disinhibition being associated with reduced LC appeal ratings in women (Figure 2A). Additionally, higher ratings of desire to eat LC food images were significantly associated with higher VAS hunger and increased food cravings (Table 3; see Figure 2B), as well as reduced VAS satiety (Figure 2C) and increased anxiety (Figure 2D), but these effects were not significant for LC appeal ratings.

### 3.4. High-Calorie vs. Low-Calorie Food Rating Differences

After adjusting for age and sex, the difference in HC and LC ratings (HC–LC) of both appeal and desire to eat was significantly positively associated with VAS hunger, disinhibition, trait-based hunger, and food cravings (Table 4). A shift from more negative to positive numbers here indicates a shift from LC > HC to HC > LC; i.e., greater hunger, disinhibition, and cravings were associated with HC foods being rated higher than LC foods.

## 4. Discussion

The goal of this study was to investigate sex-based differences in ratings of food appeal and desire to eat, for both high-calorie (HC) and low-calorie (LC) visual food cues, and to determine sex effects on key factors relevant to those ratings. We hypothesized that eating disorder-related behaviors would be more prevalent in women compared to men and, as such, would be more likely to be associated with ratings of both appeal and desire in women, particularly for HC foods. Although women did report more eating disorder-related behaviors than men (total scores on the EAT-26), relationships between this factor and ratings of food appeal and desire were not observed, nor were sex-based differences in these relationships. A possible reason for this could be that the current sample did not report high rates of eating disorder-related behaviors, and that these relationships, and the impact of sex, may be more prominent in individuals with greater reported eating disorder-related behaviors or clinical diagnoses of eating disorders [8].

We also hypothesized that rates of dietary restraint would be higher in women and that greater restraint would be associated with reduced desire to eat HC foods. Indeed, higher scores on all three subscales of the TFEQ were observed in women compared to men, including restraint, which is concordant with previous studies [26,53]. Relationships between dietary restraint and ratings of food appeal and desire were not observed, however, nor were sex-based differences in these relationships. These findings suggest that dietary restraint, which reflects the intention to control and restrict food intake for the purposes of weight control [44,54], may have a minimal impact on food ratings. Previous findings have been mixed regarding relationships between restraint and food preferences/intake, but few have focused on how these relationships may differ by sex [40,55,56]. Higher scores on TFEQ disinhibition and trait-based hunger subscales were both associated with increased ratings of HC appeal and desire to eat, although sex differences in these relationships were not observed. These effects were not significant for LC desire, which fits with a previous study from our group, in which higher disinhibition was associated with increased desire to eat discretionary foods, but not vegetables [30]. LC appeal was, however, associated with disinhibition, possibly driven by a negative relationship in women (i.e., reduced LC appeal with increased disinhibition), although a significant sex-based interaction was not observed.

We hypothesized that mood measures (depression, anxiety, stress) would be more likely to be associated with food ratings in women compared to men. Higher depression (CESD-R) and stress (PSS) scores were reported in women compared to men, consistent with previous studies [8,13]. There was a trend towards a sex difference in the relationship between HC appeal and perceived stress, with HC appeal increasing as perceived stress increased in men, but decreased in women. As this interaction did not survive multiple comparisons correction, however, this observation should be interpreted with caution. For LC foods, a trend towards increased stress being associated with increased appeal across both men and women was observed, but this effect also did not survive multiple comparisons correction. This may suggest sex-based differences in the effects of stress on the appeal of HC foods and not LC foods, but as findings did not surpass the stringent significance thresholds employed in the current study, further investigation is warranted. Previous studies suggest that stress intensity can impact the directionality of stress effects on food intake, with severe stress associated with reduced intake and mild or moderate stress with increased intake [8,57]. Stress duration, in conjunction with severity, is also important to consider, with previous work finding differential effects of acute and chronic stress on eating behaviors [8,58]. Sex differences in stress responsivity have been observed across a wide range of factors, such as hypothalamic-pituitary-adrenal (HPA) axis responsivity, appetite-related peptide expression (e.g., ghrelin, orexin), and behavioral responses to stress (e.g., coping strategies; development or exacerbation of affective disorders) [8,13]. How sex-based differences in stress responses relate to food cue responsivity and eating behaviors, however, remains unclear. A limitation of the current study is that we measured perceived stress within the past week, but did not assess factors underlying stress levels, such as types of stressors, duration of stress, and stress intensity. As all of these can impact how stress affects eating behaviors (i.e., increased vs. decreased food interest and/or intake), future studies should investigate how sex differences in response to stressors of varying intensity and duration impact eating behaviors, including how resilience to stress may differentially impact eating behaviors in men and women. While findings support future study of sex-based differences in the effects of stress on food appeal, anxiety scores were significantly associated with food *desire* in the current study, although in a similar fashion across both men and women. Increased anxiety was associated with greater ratings of desire to eat both HC and LC foods, an effect not observed for appeal ratings. Although not significant following multiple comparisons correction, there was a trend towards a similar effect for depression, with increased depression scores associated with greater HC and LC desire ratings, but not appeal ratings. Together, these results suggest a possible relationship between stress and food ratings, particularly appeal, that may be sex-dependent, while effects of anxiety and depression on food ratings, particularly desire, may be more likely to be similar across men and women.

Previous studies have observed relationships between increased BMI and higher ratings of food image appeal or desire to eat [30,59,60], but how this may differ by sex remains poorly understood due to the limited number of studies that have investigated sex-based differences in food responsivity or preferences within the context of obesity [10,25,61]. Based on previous work [30,41,59,60], we hypothesized that BMI would be associated with higher ratings of HC appeal and desire, as well as the HC–LC discrepancy, and that this relationship would be more prominent in women than men. Additionally, we hypothesized that greater HC–LC discrepancies would be associated with increased hunger, disinhibition, and cravings, and that these associations would be more prominent in women than men. Significant relationships between BMI and food ratings in the current study, however, were not observed. Additionally, sex-based differences in the relationships between BMI and food ratings were also not observed. While increased hunger, disinhibition, and cravings were indeed associated with increased HC–LC discrepancies, for both appeal and desire, these relationships also did not differ by sex. We did observe greater familiarity with LC foods in women compared to men, as well as greater familiarity with LC foods compared to HC foods, while men reported similar familiarity for both HC and LC foods. Men also rated HC desire and appeal as greater than LC desire and appeal, differences not observed in women. Additionally, women rated LC appeal higher compared to men, a difference not observed for HC appeal. This is concordant with previous work, in which women have been observed to prefer healthier foods compared to men [9,12].

Across sex, food appeal ratings were greater than desire to eat ratings, for both HC and LC foods. A previous study by our group, which used a different set of food images to investigate effects of portion size on food appeal and desire to eat, similarly found ratings of food appeal to be greater than ratings of desire to eat [30]. It was suggested that this may be due to food appeal representing a trait-like characteristic, with desire to eat being more of a state-based characteristic, such that while food may be appealing in the absence of hunger, the desire to eat the food may not be as high as it would be when hungry. As the current study was conducted in a neutral state of hunger (i.e., neither fasted nor acutely fed), this interpretation also fits well with current findings. As also observed in the current study, this previous study found VAS hunger (i.e., hunger state during assessment) to be positively associated with desire to eat, with VAS satiety negatively associated with desire to eat, supporting an influence of satiety state on desire ratings. Further supporting this, VAS satiety was not associated with food *appeal* in the current study (or in our previous study), and although VAS hunger was associated with appeal in the current work, this association was only significant for HC foods and was not as robust as that observed for desire to eat. A next step will be to assess this in the fasted state. In another previous study, we observed sex differences in brain responsivity to HC food cues in the fasted state (women > men in reward-related brain regions), but not in the fed state [14]. As such, it is possible that we might observe varying sex-based differences in relationships between food ratings and VAS measures if this were to be examined in the fasted state.

While associations between eating-related behaviors and HC food ratings were largely observed for both desire and appeal, associations with LC ratings were mostly observed for desire rather than appeal. For both HC and LC desire ratings, positive associations with both VAS hunger and food cravings were observed. Similar positive associations were observed for HC appeal ratings, but not for LC appeal, such that increased hunger and cravings were associated with greater appeal of HC foods only. Interestingly, although they did not survive multiple comparisons correction, associations between LC appeal and both hunger and cravings were in the opposite direction (i.e., increased hunger and cravings, decreased LC appeal). It is possible that while increased hunger and cravings may be associated with a greater desire to eat food in general, regardless of caloric content, LC foods may seem less appealing than HC foods as hunger and cravings increase.

Strengths of this work include a focus on sex-based differences, administering the survey in a consistent satiety state across participants, and the inclusion of multiple appetite-related measures in addition to mood measures. Results should be interpreted in the context of study limitations, however. Given that the study was conducted during the height of the COVID-19 pandemic (March–December 2020), it would be helpful to assess replicability of these relationships in a future sample, as it is possible that the pandemic may have impacted food ratings and/or mood. During this time, people experienced varying stages of schedule shifts (work, school, childcare, home activities), may have experienced impacts upon their own or loved ones’ health and well-being, and may have been experiencing a variety of different stressors than usual. Based on perceived stress scores, it does appear that most participants were experiencing moderate levels of stress. Scores on the Perceived Stress Scale (PSS) range from 0 to 56, with scores 19 and higher considered to indicate “moderate perceived stress” and scores 38 and higher considered to indicate “high perceived stress.” In this sample, the average PSS score for women was 31.1 (with 100% of women scoring above 19) and the average score for men was 28.6 (with 97.4% of men scoring above 19), suggesting that most participants perceived their level of stress as being at least moderate. As such, it will be important to investigate how relationships between stress and food ratings may change with varying levels of stress. Eating habits may also have been different during the COVID-19 pandemic, particularly during the first year, as many daily living activities were disrupted or altered (e.g., work, home life, eating timing, patterns of eating outside the home vs. cooking at home, exercise, childcare, health, etc.).

Another limitation of the current study is the reliance on self-report, particularly for measures of BMI (derived from self-reported height and weight). Additionally, while we requested that participants complete the online survey within a given time frame surrounding food intake, and asked them to report the last time they ate, there was no way to confirm that the reported time was accurate. Future work can determine if the same findings are observed with in-person assessments, during which satiety state can be more strictly monitored and BMI measures can be conducted by research staff. Furthermore, as studies have suggested BMI underestimates obesity prevalence and does not appropriately account for race- and sex-based differences in fat content and distribution, future studies could include more reliable measures of adiposity, such as percent body fat [62,63]. Finally, the current study focused on a comparison of men vs. women, but this does not capture the variability that exists in gender identity, the continuum of masculinity vs. femininity, or gonadal hormone expression, factors that can also impact mood and behavior differentially [18,64]. As such, these will also be important areas of future study.

## 5. Conclusions

In conclusion, the current study found both state- and trait-based measures of eating behaviors (hunger, satiety, disinhibition, and cravings) to be associated with ratings of food appeal and desire. These findings point to the importance of assessing these behaviors in studies investigating responsivity to visual food cues. Varying effects were observed for HC and LC foods, such that significant associations were more prevalent for HC foods. As such, it is possible that collapsing results across HC and LC foods could impact findings when investigating food cue responsivity, suggesting consideration of this effect in future work. Anxiety was associated with desire ratings for both HC and LC foods, suggesting a potential influence of mood state on the response to food cues. That trends towards associations between both stress and depression scores and food ratings were also observed may further support this, but additional investigation will be needed. Although the observed sex-based differences in dietary restraint, eating disorder-related behaviors, and mood measures in the current study were consistent with our hypotheses, the absence of sex differences in the relationships between these variables and food ratings was unexpected. This may suggest that effects of these variables on food cue responses are more consequential than potential effects of sex, or could indicate that sex in and of itself is not a meaningful contributor to these effects. However, given that men and women significantly differed in these measures in the current study, future studies including groups matched for levels of restraint, eating disorder-related behaviors, and mood measures would be helpful in parsing possible sex effects from effects relating to these group differences, or in replicating the current findings. As noted above, it will also be important to determine how variations in mood state impact responsivity to food cues (e.g., using longitudinal designs) and if this might be impacted by sex. Moving forward, in developing interventions and strategic policies to mitigate and prevent obesity, a better understanding of the influence of sex will be essential. Previous studies have suggested the importance of considering sex in identifying factors that may serve as facilitators or barriers to healthy eating and physical activity engagement [65,66]. Furthermore, the overall consideration of sex and gender in research is critical in not only understanding impacts on health behaviors and outcomes, but in driving optimization of interventions and health promotion policies at local and global levels [67,68,69,70].

## Figures and Tables

**Table 1:** Participant characteristics and behavioral measures.

	Group		Difference
Measure	Women (*n* = 155)	Men (*n* = 151)	*p* ^1^
Age (years) ^2,3^	44.9 ± 15.6	49.5 ± 14.0	**0.007**
BMI (kg/m^2^) ^2,3^	28.6 ± 7.8	29.3 ± 5.7	0.358
Race, *n* (%) ^4^			0.541
White	140 (90.3)	143 (94.7)	
Asian	7 (4.5)	2 (1.3)	
Black or African American	4 (2.6)	3 (2.0)	
American Indian or Alaska	1 (0.7)	1 (0.7)	
Native			
Other	3 (1.9)	2 (1.3)	
Ethnicity, *n* (%) ^4^			0.071
Hispanic or Latino	5 (3)	12 (8)	
Not Hispanic of Latino	150 (97)	139 (92)	
Time Since Last Ate (hours) ^2,3^	2.9 ± 2.0	3.0 ± 1.8	0.811
VAS: Hunger ^2,3^	38.0 ± 25.2	32.6 ± 24.7	0.061
VAS: Satiety ^2,3^	46.6 ± 23.1	48.2 ± 21.9	0.520
TFEQ: Restraint ^2,3^	10.0 ± 4.8	8.5 ± 4.5	**0.004**
TFEQ: Hunger ^2,3^	5.4 ± 3.6	4.6 ± 3.5	**0.044**
TFEQ: Disinhibition ^2,3^	8.0 ± 4.2	6.1 ± 3.6	**<0.001**
FCQ-S ^2,3^	33.1 ± 12.3	31.2 ± 11.7	0.170
EAT-26 ^2,3^	12.3 ± 10.5	8.1 ± 7.4	**<0.001**
CESD-R ^2,3^	17.7 ± 11.2	14.3 ± 10.4	**0.006**
STAI ^2,3^	34.8 ± 12.7	32.3 ± 11.8	0.082
PSS ^2,3^	31.1 ± 4.3	28.6 ± 5.0	**<0.001**
HC Familiarity ^2,3^	3.4 ± 0.3	3.4 ± 0.3	0.340
LC Familiarity ^2,3^	3.6 ± 0.3	3.4 ± 0.3	**<0.001**
HC Desire to Eat ^2,3^	40.3 ± 23.2	43.4 ± 24.0	0.250
LC Desire to Eat ^2,3^	42.1 ± 20.0	39.5 ± 19.4	0.255
HC–LC Desire to Eat ^2,3^	−1.8 ± 18.8	3.9 ± 18.7	**0.009**
HC Appeal ^2,3^	61.7 ± 16.4	64.5 ± 15.7	0.132
LC Appeal ^2,3^	63.2 ± 13.1	58.3 ± 13.9	**0.002**
HC–LC Appeal ^2,3^	−1.4 ± 18.8	6.3 ± 19.7	**0.001**

^1^ Significant *p* values in bold; ^2^ Mean ± SD; ^3^ Group differences examined using independent-samples *t*-test; ^4^ Group differences examined using chi-square test. VAS: visual analog scale; TFEQ: Three Factor Eating Questionnaire; FCQ-S: Food Cravings Questionnaire-State; EAT-26: Eating Attitudes Test; CESD-R: Center for Epidemiologic Studies Depression Scale Revised; STAI: State-Trait Anxiety Inventory; PSS: Perceived Stress Scale; HC: high-calorie food cues; LC: low-calorie food cues.

**Table 2:** Sex-based interactions and relationships between variables of interest and ratings of appeal and desire to eat for high-calorie food cues.

Measure	Rating Type	Effect on Ratings ^1,2^		Sex Interaction	
		*F*	*p* ^3^	*F*	*p* ^3^
Hunger (VAS)	Appeal	13.50	**<0.001**	0.11	0.742
	Desire	96.21	**<0.001**	0.04	0.849
Satiety (VAS)	Appeal	0.69	0.408	0.15	0.699
	Desire	20.32	**<0.001**	1.21	0.272
TFEQ: Restraint	Appeal	2.31	0.130	3.43	0.065
	Desire	1.96	0.163	1.41	0.236
TFEQ: Disinhibition	Appeal	47.45	**<0.001**	0.01	0.923
	Desire	40.03	**<0.001**	0.003	0.956
TFEQ: Hunger	Appeal	46.63	**<0.001**	0.28	0.598
	Desire	78.33	**<0.001**	0.02	0.898
FCQ-S	Appeal	28.62	**<0.001**	0.02	0.899
	Desire	137.24	**<0.001**	0.06	0.805
EAT-26	Appeal	1.83	0.177	0.27	0.604
	Desire	0.53	0.466	0.85	0.356
CESD-R	Appeal	1.15	0.283	0.20	0.653
	Desire	7.24	0.008	0.01	0.920
STAI	Appeal	2.65	0.104	0.77	0.379
	Desire	22.72	**<0.001**	0.003	0.955
PSS	Appeal	0.19	0.658	7.68	0.006
	Desire	0.02	0.880	1.42	0.235
BMI	Appeal	4.18	0.042	0.16	0.687
	Desire	1.69	0.195	1.54	0.216

^1^ Age and sex included as covariates in all models; ^2^ If *p* < 0.05 for the interaction with sex, values for the effect of each characteristic/measure on ratings are reported with the sex interaction included in the model. If *p* > 0.05 for the sex interaction, it was removed from the model (and reported values reflect the model without the interaction); ^3^ Significant *p* values in bold (with significance threshold of *p* < 0.002, following Bonferroni correction for 22 comparisons). VAS: visual analog scale; Desire: desire to eat; TFEQ: Three Factor Eating Questionnaire; FCQ-S: Food Cravings Questionnaire-State; EAT-26: Eating Attitudes Test; CESD-R: Center for Epidemiologic Studies Depression Scale Revised; STAI: State-Trait Anxiety Inventory; PSS: Perceived Stress Scale; BMI: body mass index.

**Table 3:** Sex-based interactions and relationships between variables of interest and ratings of appeal and desire to eat for low-calorie food cues.

Measure	Rating Type	Effect on Ratings ^1,2^		Sex Interaction	
		*F*	*p* ^3^	*F*	*p* ^3^
Hunger (VAS)	Appeal	6.83	0.009	0.68	0.411
	Desire	61.43	**<0.001**	3.00	0.084
Satiety (VAS)	Appeal	3.41	0.066	0.07	0.793
	Desire	29.25	**<0.001**	3.88	0.049
TFEQ: Restraint	Appeal	1.23	0.268	0.03	0.859
	Desire	0.08	0.784	0.40	0.527
TFEQ: Disinhibition	Appeal	12.15	**<0.001**	5.18	0.024
	Desire	3.53	0.061	0.86	0.355
TFEQ: Hunger	Appeal	9.03	0.003	0.005	0.946
	Desire	4.60	0.033	0.02	0.898
FCQ-S	Appeal	6.35	0.012	2.70	0.102
	Desire	40.66	**<0.001**	0.05	0.821
EAT-26	Appeal	0.64	0.423	2.39	0.123
	Desire	0.11	0.729	0.48	0.488
CESD-R	Appeal	0.001	0.979	2.78	0.097
	Desire	7.10	0.008	0.99	0.321
STAI	Appeal	1.41	0.235	3.66	0.057
	Desire	16.45	**<0.001**	0.006	0.940
PSS	Appeal	6.92	0.009	0.80	0.372
	Desire	2.19	0.140	0.01	0.929
BMI	Appeal	0.08	0.773	0.08	0.779
	Desire	0.58	0.445	1.55	0.214

^1^ Age and sex included as covariates in all models; ^2^ If *p* < 0.05 for the interaction with sex, values for the effect of each characteristic/measure on ratings are reported with the sex interaction included in the model. If *p* > 0.05 for the sex interaction, it was removed from the model (and reported values reflect the model without the interaction); ^3^ Significant *p* values in bold (with significance threshold of *p* < 0.002, following Bonferroni correction for 22 comparisons). VAS: visual analog scale; Desire: desire to eat; TFEQ: Three Factor Eating Questionnaire; FCQ-S: Food Cravings Questionnaire-State; EAT-26: Eating Attitudes Test; CESD-R: Center for Epidemiologic Studies Depression Scale Revised; STAI: State-Trait Anxiety Inventory; PSS: Perceived Stress Scale; BMI: body mass index.

**Table 4:** Sex-based interactions and relationships between variables of interest and the high-calorie vs. low-calorie difference (HC—LC) in ratings of appeal and desire to eat.

Measure	Rating Type	Effect on Ratings ^1,2^		Sex Interaction	
		*F*	*p* ^3^	*F*	*p* ^3^
Hunger (VAS)	Appeal	25.28	**<0.001**	0.10	0.753
	Desire	10.59	**0.001**	2.15	0.144
Satiety (VAS)	Appeal	4.00	0.047	0.26	0.609
	Desire	0.003	0.959	0.39	0.532
TFEQ: Restraint	Appeal	4.25	0.040	2.83	0.094
	Desire	4.79	0.029	4.69	0.031
TFEQ: Disinhibition	Appeal	43.23	**<0.001**	2.63	0.106
	Desire	33.23	**<0.001**	1.17	0.280
TFEQ: Hunger	Appeal	67.02	**<0.001**	0.16	0.687
	Desire	71.93	**<0.001**	0.09	0.760
FCQ-S	Appeal	41.46	**<0.001**	1.23	0.268
	Desire	38.93	**<0.001**	0.001	0.972
EAT-26	Appeal	0.32	0.572	0.42	0.515
	Desire	1.63	0.203	0.18	0.669
CESD-R	Appeal	0.78	0.378	0.63	0.427
	Desire	0.32	0.571	0.82	0.367
STAI	Appeal	4.88	0.028	0.37	0.543
	Desire	2.62	0.106	<0.001	0.993
PSS	Appeal	2.15	0.144	2.83	0.093
	Desire	3.02	0.083	2.53	0.113
BMI	Appeal	3.67	0.057	0.02	0.890
	Desire	5.95	0.015	0.06	0.806

^1^ Age and sex included as covariates in all models; ^2^ If *p* < 0.05 for the interaction with sex, values for the effect of each characteristic/measure on ratings are reported with the sex interaction included in the model. If *p* > 0.05 for the sex interaction, it was removed from the model (and reported values reflect the model without the interaction); ^3^ Significant *p* values in bold (with significance threshold of *p* < 0.002, following Bonferroni correction for 22 comparisons). VAS: visual analog scale; Desire: desire to eat; TFEQ: Three Factor Eating Questionnaire; FCQ-S: Food Cravings Questionnaire-State; EAT-26: Eating Attitudes Test; CESD-R: Center for Epidemiologic Studies Depression Scale Revised; STAI: State-Trait Anxiety Inventory; PSS: Perceived Stress Scale; BMI: body mass index.

## Data Availability

The data presented in this study are available from the corresponding author on reasonable request.

## Supplementary Materials

The following supporting information can be downloaded at: https://www.mdpi.com/article/10.3390/nu15030762/s1, Figure S1: Infographic presented at the start of the online survey to help convey the hunger state in which participants should complete the study; Table S1: Descriptions, caloric content (kcal per 100 g), and image set (high-calorie (HC) or low-calorie (LC)) for the images in the food pictures task.
