# Supplementary material for: Sex Differences in Effects of Mood, Eating-Related Behaviors, and BMI on Food Appeal and Desire to Eat: A Cross-Sectional Survey Study
Source: Nutrients. 2023 Feb 2;15(3):762. doi: 10.3390/nu15030762 (PMC9920270; doi:10.3390/nu15030762)
Supplement: Supplementary file 1 [file nutrients-15-00762-s001.zip › nutrients-2182694-supplementary.pdf]

Sex Differences in Effects of Mood, Eating-Related Behaviors, and BMI on Food Appeal and Desire to Eat: A Cross-Sectional Survey Study

Kristina T. Legget [1,2,*], Marc-Andre Cornier [3], Lauren Sarabia [1], Eve M. Delao [1], Susan K. Mikulich-Gilbertson [1], Crystal Natvig [1], Christina Erpelding [1], Tessa Mitchell [1], Allison Hild [1], Eugene Kronberg [1] and Jason R. Tregellas [1,2]

Supplementary Materials

Study Instructions

To complete this study, we require that participants are in a neutral state of hunger. That means that you have not eaten anything within the last 2 hours but that it hasn't been more than 3 hours since you last ate something. This applies to both meals and snacks. For example, if you had breakfast at 8:00 AM, you could begin the study any time between 10:00 and 11:00 AM. If you had a mid-day snack at 2:00 PM, then you would start the study any time between 4:00 and 5:00 PM. If you have not eaten within the time window described above, please hit the "Save and Return Later" button. You will be provided with a unique code to return to the survey; please write down this code and/or keep the tab open until you are eligible to participate. Thank you for complying with study procedures.

Please use Google Chrome to complete this study.

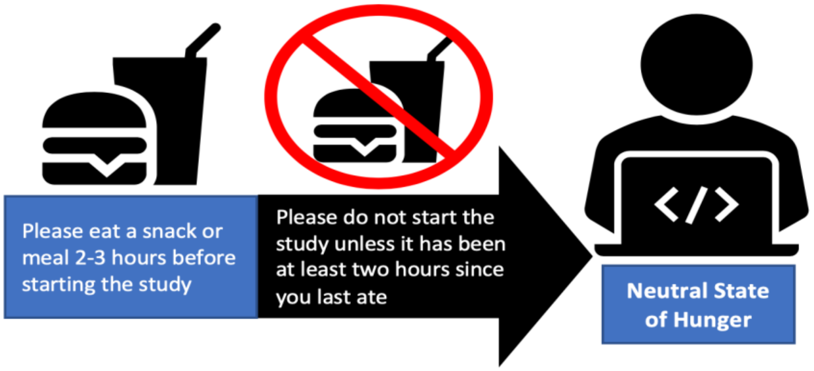

(If this is not true for you, please return to complete the study after it has been 2-3 hours since you have last eaten.)

◯ By clicking this button, you are stating that you have eaten within the time window described above and are eligible to participate at this time.

Supplementary Figure S1. Infographic presented at the start of the online survey to help convey the hunger state in which participants should complete the study.

Supplementary Table S1. Descriptions, caloric content (kcal per 100g), and image set (high-calorie (HC) or low-calorie (LC)) for the images in the food pictures task.

Description	Kcal (per 100g)	HC or LC Image Set
Ice cream sundae	279	HC
Nachos with ground beef, avocado, tomato	305	HC
"Digiorno Rising Crust" pepperoni pizza	282	HC
Pancakes with syrup	300	HC
Flourless chocolate cake with raspberries	379	HC
"Milky Way" candy bar	456	HC
"Fruit Loops" cereal with milk	163	HC
Banana split	196	HC
Oatmeal raisin cookies	450	HC
Enchiladas	142	HC
Fettuccine alfredo	387	HC
Chocolate milkshake	217	HC
Bacon	468	HC
Blueberry cheesecake	295	HC
Tater tots with ketchup	183	HC
Waffles with bacon and maple syrup	378	HC
"Carl's Jr Western Bacon Cheeseburger"	315	HC
Cream-filled cannoli with chocolate chips	471	HC
Curly fries	176	HC
Cheese pesto pizza	273	HC
Checkerboard cake with chocolate frosting	374	HC
McDonald's french fries	312	HC
Chocolate cake with frosting and cocoa powder	362	HC
Spaghetti with meatballs	143	HC
Frito pie casserole	414	HC
Baked potato with cheese, bacon, and sour cream	149	HC
"Drumsticks Lil' Drums" ice cream cone	354	HC
Key lime pie	468	HC
Assorted chocolates	441	HC
Double chocolate cake	399	HC
Waffle with ice cream and chocolate sauce	331	HC
Meat lasagna with garlic bread	202	HC
Potatoes au gratin	357	HC
Grilled cheese sandwich	364	HC
Pineapple upside-down cake	328	HC
Onion rings and "Outback Bloomin' Onion Sauce"	354	HC
Raised donuts with icing	421	HC
Vanilla cupcakes with frosting	382	HC
Cinnamon roll	452	HC
Meat lasagna	188	HC
Quesadilla with shredded pork, onions, peppers	290	HC
Red velvet cake with frosting	449	HC
Fish and chips with tartar sauce	269	HC

Peach cobbler with vanilla ice cream	204	HC
Cheeseburger	286	HC
Ice cream brownie sundae	345	HC
Potato chips with ridges	532	HC
Flatbread with cheese	262	HC
Chicken noodle soup	61	LC
Fruit platter (pineapple, grapes, strawberries, kiwi, melon)	47	LC
Orange slices	47	LC
Hard boiled eggs	143	LC
Peaches	42	LC
Watermelon (sliced)	30	LC
Grilled chicken with zucchini and squash	125	LC
Mixed berries (strawberries, raspberries, blackberries, blueberries)	46	LC
Green beans	58	LC
Omelet with mushrooms, broccolini, spinach, onions	92	LC
Fruit skewers (strawberry, orange, pineapple, kiwi, blueberry, grape)	54	LC
Kiwi	58	LC
Blackberries	43	LC
Strawberries	32	LC
Green apple slices	52	LC
Grilled zucchini	91	LC
Mixed fruit (melon, kiwi, grapefruit)	45	LC
Celery sticks (raw)	14	LC
Greek salad: cucumber, tomatoes, feta cheese, kalamata olives, red onion	91	LC
Sweet peas with butter	102	LC
Scrambled eggs with spinach and radishes	133	LC
Cauliflower (cooked)	52	LC
Pomegranate	83	LC
Beets (cooked) with Italian dressing	72	LC
Celery and carrot sticks (raw)	27	LC
Mixed greens with tomatoes	17	LC
Mixed vegetables: asparagus, tomatoes, zucchini, corn	35	LC
Green apple (halved)	52	LC
Mixed fruit (strawberries, kiwi, pineapple, cantaloupe, blueberries)	45	LC
Roasted carrots	68	LC
Sweet corn (kernels, cooked)	87	LC
Beef broth with tofu, mushrooms, celery	26	LC
Grilled chicken with asparagus	119	LC
Grapes	69	LC
Brussels sprouts (raw)	45	LC
Mixed greens with onions, bell pepper, asparagus, cucumber	20	LC
Starfruit	31	LC
Melon balls (watermelon, cantaloupe, honeydew)	33	LC
Chicken broth	6	LC
Raspberries	52	LC
Cherries	63	LC
Tilapia with collard greens and red potatoes	95	LC

Bananas (whole)	89	LC
Blueberries	57	LC
Roasted zucchini and yellow squash	51	LC
Green grapes (on vine)	69	LC
Red pear	57	LC
Cucumber (raw)	15	LC

HC: high-calorie; LC: low-calorie.
